# Unveiling the Effect of CaF_2_ on the Microstructure and Transport Properties of Phosphosilicate Systems

**DOI:** 10.3390/ma15227916

**Published:** 2022-11-09

**Authors:** Yizhe Du, Zhidan Huang, Mujun Long, Huamei Duan, Dengfu Chen

**Affiliations:** 1 Laboratory of Materials and Metallurgy, College of Materials Science and Engineering, Chongqing University, Chongqing 400044, China; 2 Chongqing Key Laboratory of Vanadium-Titanium Metallurgy and New Materials, Chongqing University, Chongqing 400044, China

**Keywords:** molecular dynamics simulation, CaF_2_, melts’ structure, transport properties, viscosity

## Abstract

As an effective flux, CaF_2_ is beneficial in improving the fluidity of slag in the steel-making process, which is crucial for dephosphorization. To reveal the existence form and functional mechanism of CaF_2_ in phosphosilicate systems, the microstructures and transport properties of CaO-SiO_2_-CaF_2_-P_2_O_5_ quaternary slag systems are investigated by molecular dynamics simulations (MD) combined with experiments. The results demonstrate that the Si-O coordination number does not vary significantly with the increasing CaF_2_ content, but the P-O coordination number dramatically decreases. CaF_2_ has a minor effect on the single [SiO_4_] but makes the structure of the silicate system simple. On the contrary, F^−^ ions could reduce the stability of P-O bonds and promoted the transformation of [PO_4_] to [PO_3_F], which is beneficial for making the P element-enriched phosphate network structure more aggregated. However, the introduction of CaF_2_ does not alter the tetrahedral character of the original fundamental structural unit. In addition, the results of the investigation of the transport properties show that the self-diffusion coefficients of each ion are positively correlated with CaF_2_ content and arranged in the order of F^−^ > Ca^2+^ > O^2−^ ≈ P^5+^ > Si^4+^. Due to CaF_2_ reducing the degree of polymerization of the whole melts, the viscosity decreases from 0.39 to 0.13 Pa·s as the CaF_2_ content increases from 0% to 20%. Moreover, the viscosity of the melt shows an excellent linear dependence on the structural parameters.

## 1. Introduction

The physical and chemical properties of slag are crucial for mass transfer and chemical reactions between liquid steel and slag. It is well-known that the physical and chemical properties of slag are determined by its structural characteristics [1,2], and it is of extraordinary interest to study the structural information of slag to understand its performance.

There is a large quantity of experimental approaches that have been applied to study the structure information of slag, and they mainly include nuclear magnetic resonance, X-ray diffraction, neutron diffraction, Raman spectroscopy, etc. [3,4,5]. These methods help one to effectively understand the microstructure and unique properties of slag, which is a significant breakthrough in this research direction. In recent years, with the rapid development of computer technology, a large number of simulation techniques have gradually entered the field of vision of scholars. In particular, MD simulations are expected to provide an effective way to understand the slag structure from a microscopic point of view with its advantages. Specifically, it is not affected by experimental conditions, such as high temperature and pressure. At present, a large number of scholars have used the molecular dynamics (MD) simulation method to conduct studies on the microstructure and properties of metallurgical slag and achieved remarkable results [6,7,8].

At present, structural information of binary and ternary silicate and aluminate melts has been extensively and carefully studied. As for phosphate melt, it has not been systematically studied due to the complexity and diversity of its structure. However, phosphorus is one of the harmful elements in steel. The excessive amount of phosphorus in steel is detrimental to its quality and properties. Therefore, dephosphorization is one of the key tasks in steel-making. In addition, phosphorus removal relies on the reaction between steel and slag, so a comprehensive study of the microstructure and transport properties of phosphorus removal slag could help clarify the underlying causes of the alteration in its macroscopic properties. In previous studies, Diao et al. [9,10] studied the microstructure of the ternary slag system of CaO-SiO_2_-P_2_O_5_ through MD simulation, and the results showed that silicon and phosphorus mainly formed a tetrahedral structure. Additionally, the concentration of free oxygen decreases significantly with increasing P_2_O_5_ content and the degree of polymerization of the melt increases. Fan et al. [11] reported the existence form of Si and P in CaO-SiO_2_-P_2_O_5_ melt under high basicity, and the results showed that both Si and P tended to form complex anions, and P ions are more inclined to form a single tetrahedron structure. Moreover, Jiang et al. [12] used MD simulation to study the structure and properties of the molten CaO-SiO_2_-P_2_O_5_-FeO slag system and concluded that the polymerization degree of the system decreased with the increase of basicity.

In our previous studies, a relatively deep understanding of the structural properties of binary phosphate systems has been obtained [1]. In actual production, additional components will be used to adjust the comprehensive physicochemical properties of the slag to meet the requirements of the metallurgical production process. CaF_2_ is widely used as a conventional flux to reduce the viscosity of slag and improve its mobility. At present, some scholars have carried out studies on the structural properties of CaF_2_-containing glasses. For example, Kansal et al. [13] studied the effect of the CaO/MgO ratio on the structure and thermal properties of CaO-MgO-SiO_2_-P_2_O_5_-CaF_2_, and found that CaF_2_ always tends to combine with [PO_4_] to form composite structures. Pedone et al. [14] investigated the influence of halides on the structure of phosphosilica bioactive glasses by MD simulation. The results show that in the mixed-fluoride/chloride-containing glasses, fluorine tends to surround phosphate, whereas chloride moves toward the silicate network. Furthermore, interestingly, according to relevant reports, F^−^ can also directly participate in the dephosphorization reaction, thus directly affecting the structure of dephosphorization slag [15,16]. However, no reports have been found regarding the presence of CaF_2_ in dephosphorized slag and its effects on structure and properties.

Therefore, to shed light on the morphological and functional mechanisms responsible for the presence of CaF_2_ in dephosphorized slag, in this paper we focus on the quaternary slag system CaO-SiO_2_-CaF_2_-P_2_O_5_ and perform a comprehensive analysis of its microstructure using molecular dynamics simulations. Under the conditions of certain contents of (xCaO)/(xSiO_2_) and P_2_O_5_ in the slag, the influence of CaF_2_ on the microstructure and transport performance of the slag system under high temperature is investigated combined with experiments. The results of this investigation can provide some valuable information to understand the microstructure of dephosphorized slag and clarify the intrinsic link between melt flow properties and the evolution of structural units.

## 2. Computational Methodology

### 2.1. Interatomic Potential

For molecular dynamics simulation, selecting appropriate potential function and corresponding parameters is the basis of accurate calculation. All molecular dynamics simulations were carried out using the Born-Mayer-Huggins (BMH) model [1,7,9,10,11] in this study, and the potential can be expressed as Equation (1):

(1) $$U_{ij}\left(r\right)=\frac{Z_{i}Z_{j}e^{2}}{4\pi \varepsilon_{0}r_{ij}}+A_{ij}\exp\left(-B_{ij}r\right)-\frac{C_{ij}}{r_{ij}^{6}}\;$$

where, *Z_i_* and *Z_j_* are the effect charges of ions *i* and *j*, respectively, *e* represents the charge of a single electron, *ε*_0_ represents the vacuum permittivity, *r_ij_* is the distance between atoms *i* and *j*, and *A_ij_*, *B_ij_*, and *C_ij_* are the adjustable parameters for BMH potentials. The three items on the right side of the above formula represent the coulombic interaction, short-range repulsion interaction, and the van der Waals interactions. Various interaction potential parameters between the selected particles are listed in Table 1 [11,17,18].

### 2.2. Simulation Approach

**Figure 1:**
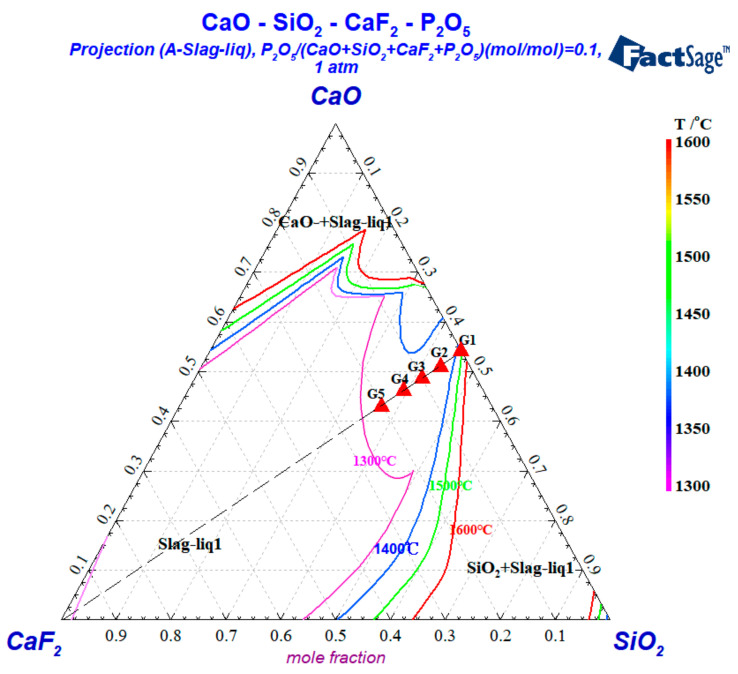
Liquid phase interval of the CaO-SiO_2_-CaF_2_-P_2_O_5_ system.

The target sample composition was discussed in the full melting component range at the corresponding temperatures. The slag samples of CaO-SiO_2_-CaF_2_-P_2_O_5_ were divided into five groups, where the first group with 0% CaF_2_ was used as a comparison with the other four groups. As Figure 1 shows, according to the liquid phase range of the CaO-SiO_2_-CaF_2_-P_2_O_5_ quaternary slag system at 1600 °C obtained through Factsage 8.0 thermodynamic calculation software, the chemical composition of each group in this study was determined. The varying number of atoms was then calculated based on the mole fraction of each group. Referring to the empirical formula in relevant literature and research results [19], the density of various groups at 1600 °C was calculated, respectively. The chemical composition, atomic number, and other information about each group of samples are listed in Table 2.

The computational methods used and the choice of parameters are critical factors in achieving efficient and accurate simulations. In this study, about 6000 atoms were randomly placed in a model box. Since the number of calculated atoms is always finite, periodic boundary conditions were performed on all faces of the model box to obtain an infinite system of atoms without boundaries. The results obtained with periodic boundary conditions are sufficient to reflect the actual situation. All MD simulations use a canonical ensemble (NVT), which means that the calculations were performed in a system with a constant atomic number (N), sample volume (V), and temperature (T). Additionally, the sum method of Ewald was used for the long-distance coulomb force, and the motion equation of atoms was explained by the jump integral method of a 1 fs timestep. The potential cutoff radius was set to 10 Å in the calculation of the repulsive force. Besides, the total time length of each group of simulations was determined to be 60 ps, equivalent to 60,000 steps. After the beginning of the simulation, the initial temperature was set at 5000 K (4727 °C) for 15,000 timesteps to agitate the atoms and eliminate the effect of the intentional distribution. Secondly, the temperature was cooled down to 1873 K (1600 °C) with 30,000 timesteps. Subsequently, the system was relaxed at 1600 °C for another 15,000 timesteps in the equilibrium calculation. The temperature, volume, and enthalpy remained nearly constant for 15,000 timesteps, demonstrating that the system has reached equilibrium.

### 2.3. Statistics of Structural Information

The radial distribution function (RDF) is commonly used to investigate the character of the short-range order of melts. Equation (2) lists the mathematical expression of the RDF [20]:

(2) $$g_{ij}\left(r\right)=\frac{V}{N_{i}N_{j}}\underset{j}{\sum }\frac{\langle n_{ij}\left(r-\Delta r/2,r+\Delta r/2\right)\rangle }{4\pi r^{2}\Delta r}$$

where, *V* is the volume of MD-simulated cells, *N* is the number of particles, and $n_{ij}$ is the average number of atom *j* surrounding the atom *i* within a distance *r* ± Δr/2. The abscissa of the first peak and the first trough in the RDF curve represent the average bond length and cutoff radius of the corresponding atoms, respectively.

In addition, integration of the corresponding partial RDF of the particles generates the average CN function, which represents the number of atoms *j* around atoms *i* within the cutoff radius. The CN function is expressed as Equation (3):

(3) $$N_{ij}\left(r\right)=\frac{4\pi N_{j}}{V}\int_{0}^{r}r^{2}g_{ij}\left(r\right)dr\;$$

Finally, as far as the concentration of oxygen species and the distribution of structural units, Q^n^, were concerned, this structural information will be counted by the Matlab program, based on the spatial atomic coordinates derived from the MD simulation.

### 2.4. Viscosity Calculation

Viscosity is one of the most significant physical parameters of slag. The viscosity of melts can reflect the degree of polymerization. Through statistical analysis of atomic coordination by MD simulation, a function of *MSD* would be generated, displayed as Equation (4):

(4) $$MSD=\langle \Delta r{\left(t\right)}^{2}\rangle =\frac{1}{N}\langle \overset{N}{\underset{i=1}{\sum }}{\left|r_{i}\left(t\right)-r_{i}\left(0\right)\right|}^{2}\rangle$$

where, *N* is the number of particles, $r_{i}\left(t\right)$ represents the position coordinate of atom *i* at time t, and angular brackets denote a statistical average of many function values. The self-diffusion coefficient could be obtained from *MSD* as shown below [21]:

(5) $$D=\underset{t\to \infty }{lim}\frac{1}{6}\frac{d\left[\Delta \bar{r}{\left(t\right)}^{2}\right]}{dt}$$

Then, the shear viscosity information of the melts can be obtained by combining the self-diffusion coefficient, *D,* with the Stokes–Einstein equation [22,23]:

(6) $$\eta =\frac{K_{B}T}{D\lambda }\;$$

where, $K_{B}$ is the Boltzmann constant, which is 1.38 × 10^−23^ J/K, T is the system temperature, and λ is the particle transition step size, which is commonly considered λ = 2R_O_ = 2.8 Å [24,25,26]. Based on the above calculation method, the partial transport performance of the melts can be obtained, and the relationship between the structural information and performance can be established.

## 3. Experimental Method

Based on the mole fraction of each sample in Table 2, the composition of the experimental slag was obtained by mass conversion. The results are shown in Table 3. The reagents used in our experiments are all from a specialist chemical reagent company in Chongqing, China. The purity of the reagents (CaO, SiO_2_, CaF_2_, and P_2_O_5_) used was above 99.5 wt.%. The weighted sample powder was well-mixed and placed in a graphite crucible before the viscosity was measured.

The viscosity was measured using the rotating cylinder method. The viscometer was calibrated at room temperature using an oil with known viscosity prior to the experiment. Approximately 250 g of each slag sample was placed into a graphite crucible to melt, and the average heating rate was 5 °C/min. Since P_2_O_5_ has a low boiling point, it is prone to volatilization and produces white smoke at high temperatures. Consequently, the other three components were firstly added into the crucible and heated to 1500 °C for 20 min. After the sample was fully melted, P_2_O_5_ was added, and the crucible was covered to prevent volatilization. Then, the crucible was opened after keeping it for 5 min. If there was no obvious white smoke, the crucible was reheated to 1600 °C and kept for 20 min to homogenize the chemical composition. Finally, the viscosity of each sample was determined from an average of 60 consecutive measurements.

## 4. Results and Discussion

### 4.1. Local Structural Characteristics

**Figure 2:**
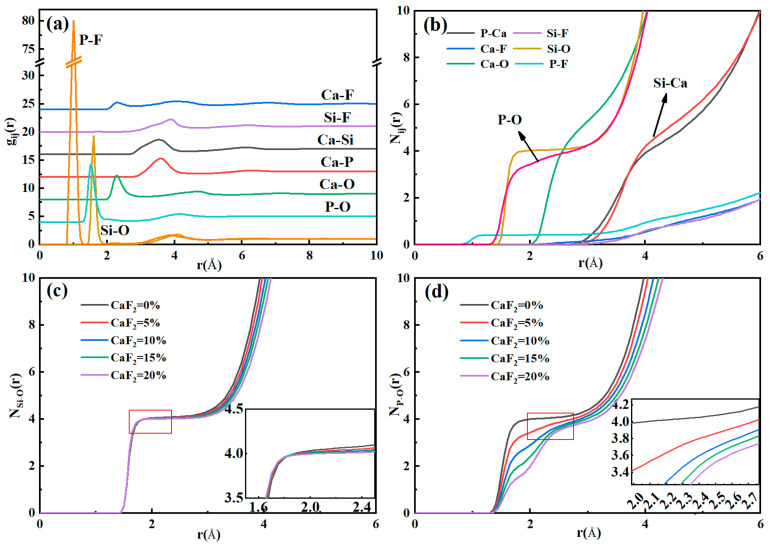
(**a**) RDF and (**b**) CN for different atom pairs in CaO-SiO_2_-CaF_2_-P_2_O_5_ systems and changes of P-O and Si-O coordination numbers: (**c**) Si-O and (**d**) P-O.

The local structure information of melts can be preliminarily obtained by RDF and CN. Taking G2 as an example, Figure 2a,b show the distribution of RDF and CN in the system of CaO-SiO_2_-CaF_2_-P_2_O_5_ at 1600 °C when CaF_2_ content was 5%, respectively. According to the RDF curve, the average bond length of each atom pair in the melts can be concluded. As can be obtained from Figure 2a, the average bond lengths of Ca-O, Si-O, P-O, and Ca-F were 2.31, 1.62, 1.50, and 2.30 Å, respectively. The results are in good accordance with previous research obtained from MD simulations and experiments [9,10,11,27]. Table 4 shows the variation of the bond lengths for various pairs of atoms from G1 to G5.

In general, a strong and sharp peak in the RDF curve indicates a steep stabilization of the corresponding bond. Similarly, for CN curves, a broad flat plateau implies a large stability of the corresponding polyhedron. It can be observed in Figure 2a that both Si-O and P-O curves had a sharp peak, meaning Si and P tended to combine with O atoms and form stable structures. From Table 4, the average Si-O bond length remained constant with the increasing CaF_2_ content in the melt, demonstrating that the Si-O bond was particularly stable and was not subject to CaF_2_. However, the length of the P-O bond became longer, confirming the character of the P-O structure affected by CaF_2_, while indicating a decrease in the strength of the P-O bond, which may lead to the evolution of the phosphate melt structure. Furthermore, the P-F bond appeared in the system due to the addition of CaF_2_. Interestingly, the RDF curve for the P-F bond had an unusually sharp peak and the P-F bond length did not change significantly with CaF_2_ content, indicating that the P-F bond was considerably more stable than the P-O bond, which is unprecedented. In addition, the Ca-O and Ca-F bonds had slightly increased lengths, indicating that they were more loosely bound with the addition of CaF_2_.

As can be seen from Figure 2b, CN_Si-O_, CN_P-O_, and CN_Ca-O_ were 4.04, 3.87, and 5.51, respectively. The plateau on the CN_Si-O_ curve was smoother than that on the CN_P-O_ curve, indicating that the stability of the Si-O structure was higher than that of the P-O in the CaO-SiO_2_-CaF_2_-P_2_O_5_ systems. Since Ca^2+^ is typically present as a network modifier, CN_Ca-O_ exhibited a sloping plateau, meaning that no stable structure was formed between Ca-O, which is consistent with previous studies on slag or glassy structures containing CaO [10,28,29,30]. Additionally, it is worth noting that CN_P-F_ had an extremely flat plateau between 0 and 1, which means that F^−^ and P^5+^ had a strong coordination tendency. Wang et al. [31] introduced CaF_2_ into CaO-SiO_2_-Al_2_O_3_ slag systems and found that F^−^ has a strong tendency to replace an O in the [AlO_4_] structure to form an Al-F bond. They attributed the phenomenon to the difference between the electronegativity of F^−^ and O^2−^. Therefore, the addition of CaF_2_ causes a shift in the original structure of the melts, especially for phosphate systems. The tendency of F^−^ to coordinate with P^5+^ is so strong that it may form a competitive relationship with O^2−^, leading to a large-scale transformation of the P-O structure. This trend may be more significant in high-temperature conditions. Besides, the CN curves for all pairs of atoms except Si-O, P-O, and P-F did not have a clear plateau, suggesting that they do not typically form stable structures, and they are therefore not discussed in detail here.

Figure 2c,d show the alters of Si-O and P-O coordination numbers as CaF_2_ content in slag from 0% to 20%, respectively. At 0% CaF_2_ content, the coordination numbers of Si-O and P-O were close to 4.0, indicating that most of them exist as 4-coordinates and conform to the tetrahedral form. From Figure 2c, the coordination number changes of Si-O were not obvious in the range of the mole fraction of (CaF_2_) = 0~20%, which were all around 4.0. Due to the high stability of [SiO_4_], it is difficult for F^−^ to break through the bond energy barrier between Si-O to coordinate with Si^4+^, which was also discussed in previous studies [32,33]. However, when increasing the CaF_2_ content from 0% to 20%, the coordination plateau of P-O became increasingly tortuous and the average coordination number decreased, with values of 4.05, 3.87, 3.74, 3.66, and 3.57. Moreover, it can be seen from the variation law of the bond length that P-O kept increasing, indicating that its stability decreased. In contrast, the P-F bond length was much smaller than the P-O bond length. All indications show that the affinity between P^5+^ and F^−^ is greater than that of O^2−^, which confirms that CaF_2_ will affect the coordination of P-O and alter the original [PO_4_] structure.

**Figure 3:**
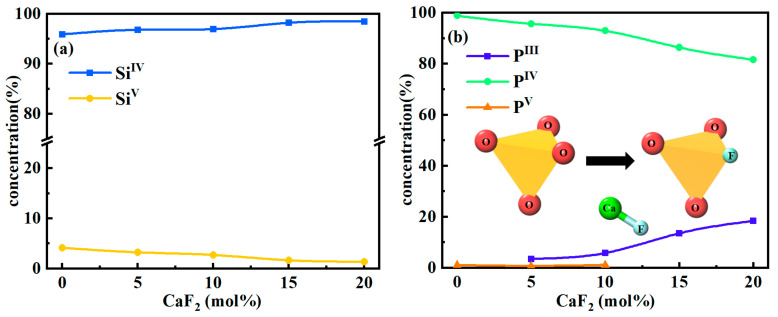
Coordination of Si-O and P-O under different CaF_2_ contents: (**a**) Si-O and (**b**) P-O.

Figure 3 shows the coordination distributions for Si-O and P-O, with superscripts indicating coordination numbers. The content of SiI^V^ was always above 95%, which shows that [SiO_4_] is the main structural unit in silicate systems and the content of [SiO_4_] did not alter significantly with the increase of CaF_2_ content, which is consistent with the findings of Fan et al. [34]. From Figure 3b, when the CaF_2_ content was 0, virtually all P-O in G1 appeared in a 4-coordination structure, indicating that the majority of P exists in slag in the form of a [PO_4_] structure and serves as the basic structural unit of the phosphate systems. However, as the CaF_2_ content increased, the PI^V^ content decreased and the P^V^ gradually disappeared, while the PIII content continued to increase. Therefore, in contrast to silicate systems, the structural units of phosphate systems absolutely change with increasing CaF_2_ content, and new structures may emerge as the coordination number of P-O gradually evolves from high to low. With the gradual increase of tri-coordinated P content, combined with the coordination of P-F in Figure 2b, it indicated that the addition of CaF_2_ prompted F^−^ to replace O^2−^, and a [PO_4_] to [PO_3_F] structural transition occurred. A similar phenomenon also appeared in the study of the phosphate glass structure by Rao et al. [35] and Touré et al. [36]. However, in Pedone et al.’s [14] work, no P-F/Cl bonds were found at room temperature. It may be that the particles become more active and their diffusion ability is enhanced at high temperatures compared with normal temperatures, which provides favorable thermodynamic and kinetic conditions for the bonding between P^5+^ and F^−^.

### 4.2. Distribution of Bond Angles

**Figure 4:**
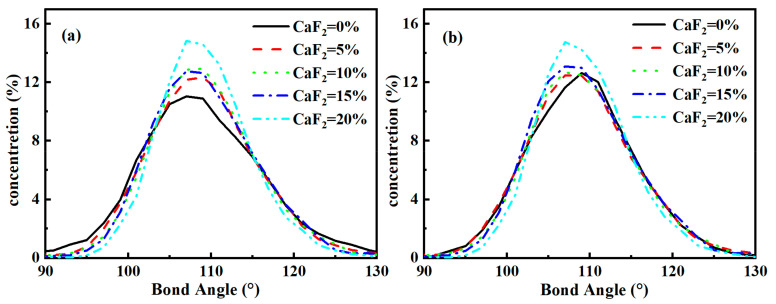
Distribution of bond angles in the system of varying CaF_2_ content: (**a**) O-Si-O and (**b**) O-P-O.

The distribution of bond angles is also a critical parameter to characterize the structure of the melt. Figure 4 shows the statistics of the O-Si-O and O-P-O bond angles’ information with varying CaF_2_ content. CaF_2_ had a negligible effect on the distribution of O-Si-O and O-P-O bond angles in the CaO-SiO_2_-CaF_2_-P_2_O_5_ system. When CaF_2_ content increased from 0% to 20%, the average bond angles of O-Si-O and O-P-O were 109.2° and 108.7°, respectively, which are very close to the theoretical value of 109.5° of standard tetrahedron. It indicates that although F^−^ tended to replace the position of an O^2−^ in [PO_4_] tetrahedron, it did not affect some structural characteristics of the original P-O bond, and the network structure with Si^4+^ and P^5+^ as the core still maintained the tetrahedral structure. Moreover, CaF_2_ did not appear to cause large-scale rearrangements of the atoms in the whole systems, which consisted of a polymeric tetrahedral structure of Si^4+^, P^5+^, F^−^, and O^2−^, as well as network modifiers such as Ca^2+^ dispersed.

### 4.3. Structural Unit Evolution

**Figure 5:**
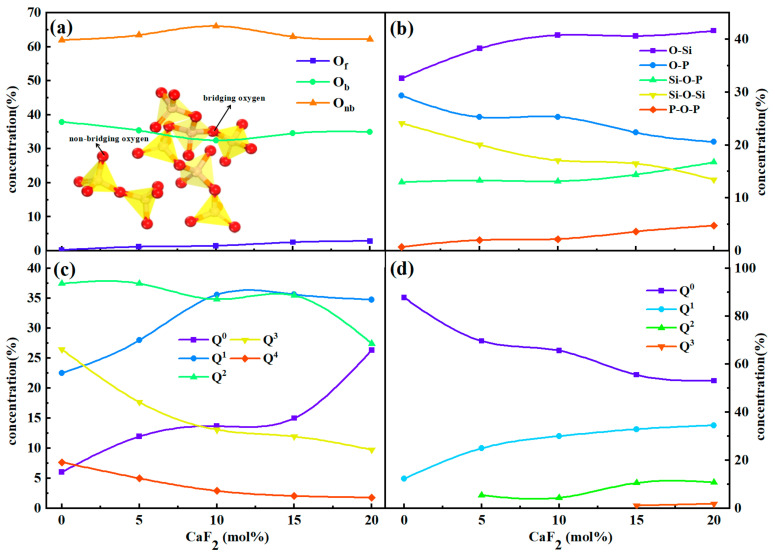
Structural unit evolution. (**a**) Types of oxygen atoms and (**b**) types of oxygen atoms after subdivision. (**c**,**d**) Distribution of Q^n^ with different CaF_2_ contents: (**a**) Q^n^Si and (**b**) Q^n^P.

The silicate and phosphorene systems mainly consist of a network structure with O atoms connected to Si and P atoms. There are three types of distinct oxygen, which are divided into free oxygen (O_f_), non-bridging oxygen (O_nb_), and bridging oxygen (O_b_). Additionally, a unique tri-coordinated oxygen structure has been found in the aluminate system according to the literature [7]. Bridging oxygen with two tetrahedra, including Si-O-Si, Si-O-P, and P-O-P, improved the degree of polymerization of the system. Non-bridged oxygen was attached to only one tetrahedron, namely O-Si and O-P, while the other end was attached to a metallic cation. They function in the opposite way to bridging oxygen. The free oxygen is not connected to any tetrahedron. The cutoff radii of Si-O and P-O were selected to be 2.3 and 2.5 Å, respectively, and the distribution of various oxygen types in the melts was collected in Figure 5a. With the increase of CaF_2_ content, the amount of free oxygen in the melts slightly increased, while the shift of the number of bridging oxygen and non-bridging oxygen had no obvious rule and was approximately in dynamic equilibrium.

O_b_ and O_nb_ were further subdivided in the melts, as shown in Figure 5b. There are three types of O_b_: Si-O-Si, Si-O-P, and P-O-P. As the CaF_2_ content increased, Si-O-Si decreased from 24.1% to 20.0%, Si-O-P increased from 13.0% to 16.7%, and P-O-P increased from 0.7% to 4.8%. It has been shown that CaF_2_ is beneficial in disrupting Si-O-Si and losing the initially polymeric silicate network structure. The Si-O-P structure in the system increased; that is, the addition of CaF_2_ promoted the connection between [SiO_4_] and [PO_4_] or [PO_3_F], resulting in a silicophosphate composite structure that was more easily established in the systems. Moreover, the increase of P-O-P also indicates that the connectivity of the phosphate network structure became higher, which makes the phosphate melt structure more complex.

To further quantitatively analyze the influence of CaF_2_ on the network structure of the systems, Q^n^ was introduced to characterize the polymerization degree of silicate and phosphate systems respectively, where n represents the number of bridging oxygen (O_b_) in a single tetrahedral unit. The current results show that Q^n^ can be classified into five types: Q^0^, Q^1^, Q^2^, Q^3^, and Q^4^, indicating that 0, 1, 2, 3, and 4 O_b_ are connected in a tetrahedral element. Figure 5c,d show the distribution of Q^n^ in silicate and phosphate systems, respectively. As CaF_2_ content increased, the Q^0^ and Q^1^ in the silicate system increased, while the Q^2^, Q^3^, and Q^4^ decreased, again confirming that CaF_2_ breaks the high connectivity between [SiO_4_] tetrahedral structures, simplifying the structure of the silicate systems. Besides, only Q^0^ and Q^1^ structures originally existed in the phosphate system, indicating that [PO_4_] normally exists in the form of a single tetrahedron or pairings, which is consistent with the research results of Fan et al. [11]. However, as the CaF_2_ content increased, the Q^0^ rapidly decreased and the Q^1^ increased. In addition, several Q^2^ and Q^3^ structures appeared and continued to increase. The results indicate that the original phosphate structure was not complicated, and the connectivity between the [PO_4_] tetrahedra was low. However, the addition of CaF_2_ reduced the number of single [PO_4_] tetrahedral elements and the current [PO_3_F] structure tended to form a chain or network composite structure, which increased the connectivity of the phosphate network. Macroscopically, higher connectivity is beneficial to the enrichment of P elements. In other words, CaF_2_ can enrich the phosphate network, which is favorable for dephosphorization.

### 4.4. Transport Properties and Viscosity

**Figure 6:**
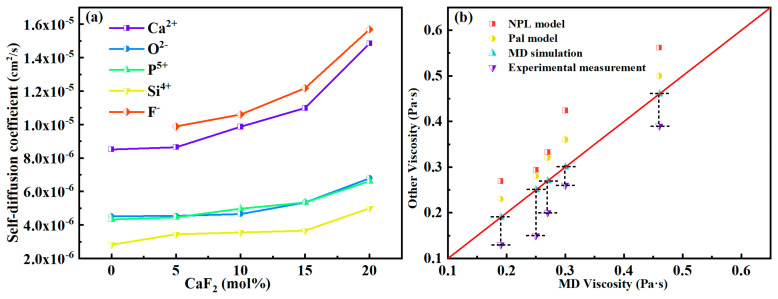
(**a**) The self-diffusion coefficient of each atom with different CaF_2_ contents. (**b**) Viscosity comparison of MD simulation, models, and experimental measurement.

The above results indicate that increasing the CaF_2_ content simplified the structure of the silicate system in CaO-SiO_2_-CaF_2_-P_2_O_5_ melts but complicated the structure of the phosphate system. Therefore, to further understand the effect of CaF_2_ on the degree of polymerization of the whole melt and to assess the changes in macroscopic properties, it is necessary to quantitatively analyze the transport properties of the system. Liquid molecules do not stay in a fixed position but are constantly moving [37]. The self-diffusion coefficient is a momentous parameter that reflects the diffusivity of the particles in the melt. As shown in Figure 6a, based on the *MSD* function and the Einstein relation, the self-diffusion coefficients of distinct ions can be obtained. It can be seen that the order of the self-diffusion coefficients of different ions was F^−^ > Ca^2+^ > O^2−^ ≈ P^5+^ > Si^4−^, and they were all in direct proportion to the content of CaF_2_, indicating that the addition of CaF_2_ can make each ion become more active. It mainly results from the depolymerization of the network structure in the melt by CaF_2_, which lowers the energy barrier for the migration of ions in the melt and enhances the mobility of each particle. In addition, these phenomena can also lead to changes in the macroscopic properties of the melts. It is worth noting that the diffusion capacity of F^−^ in the melts was most prominent and much larger than that of O^2−^, indicating that the substitution of F^−^ for O^2−^ improved the overall mobility of the phosphate structural units. Moreover, the diffusion coefficients of P^5+^ and O^2−^ were equivalent, which means that P and O always maintained the stable structure of [PO_4_] or [PO_3_F] and diffused cooperatively throughout the melts.

The melts’ viscosity was calculated from the self-diffusion coefficient of each ion and compared with the experimental measurements. The results of the MD simulation and experiment in Figure 6b both show that with the increase of CaF_2_ content, the viscosity of the CaO-SiO_2_-CaF_2_-P_2_O_5_ systems decreased and led to an improvement of melt liquidity. Clearly, the viscosity, which reflects the viscous resistance of the melt during the flow and depends prominently on the degree of polymerization of the melt, would be reduced in a melt with simple structural units. Besides, the NPL model [38] and Pal model [39] were also used to compare the calculation results. It can be observed that although there were some errors between the calculated viscosity consequences and the experimentally measured ones, the trends were in perfect agreement, which indicates that the MD simulations were able to predict the viscosity of the system accurately to some extent and reflects the reliability of the MD simulations. The predictions of both models differed significantly from the experimental data due to discrepancies in some of the components. Consequently, the MD viscosity calculations are in better agreement with the experimental results compared to both models.

In the process of steel-making dephosphorization, P is usually enriched in 2CaO·SiO_2_-3CaO·P_2_O_5_ (C_2_S-C_3_P) solid solution [40,41,42]. Dephosphorization depends on the concentration of phosphorus in the solid solution, and the flow properties of the dephosphorized product in the slag also determine whether phosphorus can be efficiently removed from the slag. As can be seen from the above analysis, the introduction of CaF_2_ directly changed the basic structural units of the phosphate melt, making the phosphate network units more easily enriched. On the other hand, CaF_2_ reduced the viscosity and improved the fluidity of the slag, so that the dephosphorized products enriched in P could be better transported to and removed from the slag layer. CaF_2_ is thus favorable for dephosphorization both from the microscopic reaction point of view in slag and from the macroscopic flow properties. Our study links the microscopic to the macroscopic and essentially defines the critical role of CaF_2_ in the dephosphorization of slag.

### 4.5. Correlation between Viscosity and Structural Properties

**Figure 7:**
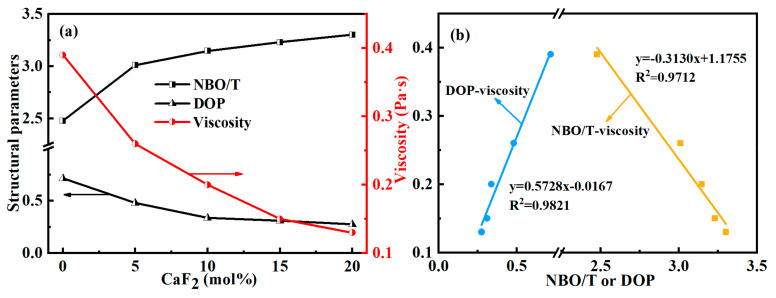
(**a**) Change of structural parameters and viscosity and (**b**) the relationship between viscosity and structural parameters.

The viscosity of the slag depends on its degree of polymerization. Researchers have proposed two common approaches to describe the complexity of melts. The first one amounts to counting the number of non-bridged oxygen atoms, denoted as NBO, based on the results of molecular dynamics simulations. The parameter NBO/T, which reflects the degree of melts’ polymerization, can be obtained by combining the number of network formers, T (Si or P), in the system [43]. The larger the NBO/T, the higher the ratio of non-bridging oxygen in the melts, that is, the simpler the structure of the melts is, then the viscosity and other parameters of the melts will also change accordingly. The second is to judge the melts’ complexity according to the evolution of total Q^n^. It is usually expressed by the ratio of high-complexity Q^n^ to low-complexity Q^n^, such as, DOP = (Q^3^ + Q^4^ + Q^5^)/(Q^0^ + Q^1^ + Q^2^). [44]. The higher the DOP, the more complex the systems. Comparing the calculated melts’ viscosity with the above two parameters, we observed a correspondence between the viscosity and the two parameters, as shown in Figure 7.

It can be observed in Figure 7a that NBO/T increased with the increase of CaF_2_ content, while DOP was the opposite. The results show that CaF_2_ can effectively reduce the complexity of the system, and the variation of both quantities has a good correspondence with the trend of the viscosity value, suggesting that the melting viscosity is directly related to the complexity of the system. Specifically, the introduction of CaF_2_ simplified some complex network units formed by interweaving [SiO_4_], [PO_4_], and [PO_3_F] structures in the whole melt, and formed simple structures such as single or chain, greatly reducing the connectivity of the whole melt. Furthermore, the complexity of the slag structure depends on the competing effects of silicates and phosphates on the polymerization of the molten slag. In the CaO-SiO_2_-CaF_2_-P_2_O_5_ systems, CaF_2_ promoted the disaggregation of complex network units into small units, which made the diffusion of micro-particles easier. The macroscopic manifestation of this phenomenon is a reduction of the total viscosity.

In Figure 7b, the relationship between the viscosity and the above two parameters was obtained by linear fitting. For viscosity and DOP, y = 0.5728x − 0.0167, R^2^ = 0.9821, and for viscosity and NBO/T, y = −0.3130x + 1.1755, R^2^ = 0.9712. The correlation coefficients of the above two fitting results were high enough, so the relationship between viscosity and microstructure of CaO-SiO_2_-CaF_2_-P_2_O_5_ melts could be accurately described, and at the same time, the viscosity could also be predicted by the microstructure of the systems.

## 5. Conclusions

We have presented the microstructure information of the CaO-SiO_2_-CaF_2_-P_2_O_5_ melts at 1600 °C by MD simulation and explored the evolution of each structural unit with the increase of CaF_2_ content. Combined with the analysis of microscopic particle transport and macroscopic flow properties, it is clear that the crucial role played by CaF_2_ in phosphosilicate melts has been investigated.

By analyzing the distributional properties of the coordination and bond angles between different atoms, we found that both S^4+^ and P^5+^ were present in tetrahedral form in the molten CaO-SiO_2_-CaF_2_-P_2_O_5_ system. The coordination number of Si-O was maintained at around 4.0 when increasing the CaF_2_ content from 0% to 20%, while the coordination number of P-O decreased from 4.05 to 3.57. Therefore, CaF_2_ had little effect on the structure of [SiO_4_] but decreased the stability of the [PO_4_] structure. Specifically, F^−^ tended to replace O^2−^ and promote the transformation of [PO_4_] to a [PO_3_F] structure, and at the same time, it is beneficial to make the P element-enriched phosphate network structure more aggregated. However, the addition of CaF_2_ did not lead to a large-scale rearrangement of the atoms in the whole system, and the network structure with Si^4+^ and P^5+^ as cores remained tetrahedral.

The results of the MD simulation and experiment showed that CaF_2_ is beneficial for reducing the degree of polymerization of the melt and thereby reducing the melt viscosity, which decreased from 0.39 to 0.13 Pa·s as the CaF_2_ content increased from 0% to 20%, and it had a good linear relationship with the structural parameters. In summary, CaF_2_ is beneficial for dephosphorization both from the microscopic reaction point of view and from the macroscopic flow properties in slag.

## Figures and Tables

**Table 1:** Born–Mayer–Higgins potential parameters for atomic pairs in CaO-SiO_2_-CaF_2_-P_2_O_5_ systems.

Atom 1	Atom 2	*A_ij_* (eV)	*B_ij_* (1/Å)	*C_ij_* (eV·Å^6^)
Ca	Ca	329,051.60	6.25	4.33
Ca	Si	26,674.68	6.25	0
Ca	P	164,585.80	12.5	0
Ca	O	717,827.00	6.06	8.67
Ca	F	496,191.60	6.06	8.67
Si	Si	2162.39	6.25	0
Si	P	1081.60	12.5	0
Si	F	43,406.00	6.06	0
Si	O	62,794.37	6.06	0
P	P	0	0.0	0
P	F	365,232.88	11.8	0
P	O	1847.70	3.45	0
F	F	730,722.80	5.88	17.34
F	O	1,046,135.40	5.88	17.34
O	O	1,497,049.00	5.88	17.34

**Table 2:** Composition, number of atoms, and density of CaO-SiO_2_-CaF_2_-P_2_O_5_ melts at 1600 °C.

Groups	Mole Fraction (%)				Number of Atoms					Total	Density (g/cm^3^)
	CaO	SiO_2_	CaF_2_	P_2_O_5_	Ca	Si	P	F	O		
G1	49	41	0	10	1010	845	412	0	3732	5999	2.5159
G2	46	39	5	10	1041	796	408	204	3551	6000	2.5109
G3	44	36	10	10	1095	730	405	405	3365	6000	2.5083
G4	41	34	15	10	1124	682	401	602	3191	6000	2.5018
G5	38	32	20	10	1152	636	397	795	3020	6000	2.4947

**Table 3:** The chemical compositions of experimental slags.

Groups	Slag Composition (Mass Percent)			
	CaO	SiO_2_	CaF_2_	P_2_O_5_
G1	41.5	37.1	0	21.4
G2	38.3	34.8	5.8	21.1
G3	36.1	31.7	11.4	20.8
G4	33.1	29.5	16.9	20.5
G5	30.3	27.3	22.2	20.2

**Table 4:** Variation of average bond lengths of different atom pairs.

Pair	Ca-O	Si-O	P-O	P-F	Ca-F	P-Ca
R_ij_/Å (G1)	2.31	1.61	1.50	—	—	3.60
R_ij_/Å (G2)	2.31	1.61	1.52	1.01	2.31	3.60
R_ij_/Å (G3)	2.31	1.61	1.54	1.01	2.31	3.60
R_ij_/Å (G4)	2.32	1.61	1.55	1.01	2.32	3.61
R_ij_/Å (G5)	2.32	1.61	1.56	1.01	2.32	3.61

## Data Availability

Not applicable.
